# Plaster, Vulcanite and Esthetics

**Published:** 1915-03

**Authors:** George H. Wilson

**Affiliations:** Cleveland, Ohio


### PLASTER, VULCANITE AND ESTHETICS.

BY GEORGE H. WILSON, D.D.S., CLEVELAND, OHIO.

Plaster of Paris is made from the mineral gypsum. The chemical composition of pure gypsum is calcium sulphate and water. Gypsum is prepared for market by grinding and burning, and mixing with accelerators, and retarders. The products of manufacture are designed for a definite purpose, and can not be interchanged advantageously.

There are requirements of practice that make a demand for as many classes of plaster: (1) for impressions the plaster should be quick setting, expand but little (not contract) and easily broken; (2) for casts the plaster should be as little changeable while crystallizing and under stress as possible; (3) for flasking and luting a medium between the preceding two is required. However, the first class of plaster can be dispensed with by adding a chemical substance to a well chosen third class plaster, and is used for impressions. A strictly impression plaster is a dangerous thing in the laboratory, as it is unfit for casts and flasking, and is too apt to be improperly used. The addition of sodium chloride or potassium sulphate to a high grade suitable plaster for flasking answers well for impressions.

If the mix of French's regular dental plaster is made, containing one ounce of water and two ounces of plaster and left exposed until it ceases to lose weight (about 25 hours), it will be found that it has lost approximately 22% of its entire weight. As the loss is the water, the 22% deducted from the one-third (33.3%) water would show approximately 11.3% had combined permanently with the plaster.

The points thus derived from this hydration experiment are: That it is impossible to mix dental plaster without an excess of water; that the larger the excess of water the less dense and weaker the plaster, and that the smaller the excess the denser and stronger the plaster. Conclusion: the plaster should be hydrated to meet the requirements of the operator.

The theory of setting of plaster is the result of two distinct series of operations that take place simultaneously, supersaturation and crystallization. The particles of calcium sulphate in the act of hydration are dissolved in the water used to gauge them, and produce a supersaturated solution; the solution thus formed deposits crystals of the hydrated sulphate. These crystals gradually increase in size, and form a compact mass in the same way as do all similar crystals deposited slowly from a saline solution, and this process continues as long as any of the more anhydrous sulphate remains available to become dissolved and to keep the solution supersaturated.

There are three properties of plaster of Paris on the workings of which the success of the dentist almost wholly depends: contraction, expansion and compression. Too much water will cause a maximum of contraction and a minimum of compression or resisting strength, while too much plaster gives resisting strength, but unfortunately a maximum of expansion.

In impression taking, a weak plaster being desired, an addition of potassium sulphate can be made to hasten the setting and reduce the expansion and strength. For use in casts plaster of Paris can not compare with the Spence Plaster Compound.

The Spence Plaster Compound changes form less than any of the pure plasters and attains its maximum strength in one hour; it is stronger in thirty minutes than a pure plaster at its maximum.

This compound is possibly four or five times as resistant to compression as any of the plaster of Paris mixes; its contraction or expansion is very small. This very useful compound is composed of plaster of Paris, Portland Cement and chemicals to control its setting and expansion properties.

The working of this compound is more laborious than mixing plaster of Paris. The ratio of water to the compound is one to four.

For a cast a fluid ounce of water is placed in the plaster bowl, and three measured ounces of the compound are added and thoroughly spatulated with a stiff spatula, until it becomes soft and plastic, after which a half ounce more may be thoroughly incorporated. The remaining half ounce may be better added one-half at a time. It must be spatulated and kneaded in the bowl until the mass is of putty-like consistence. If properly mixed it can now be taken in the fingers and squeezed to expel gases. It is now packed by degrees into the impression and jarred to fill all parts. If now turned over on a piece of celluloid it will not stick when removed.

### VULCANITE.

Vulcanite for artificial dentures is composed of carbon, 10 parts; hydrogen, 16 parts, combined with sulphur in any proportion from 1 to 20.

### THE PHYSICAL PROPERTIES.

Rubber expands more rapidly than any other solid body. Its rate of expansion at ordinary temperatures from 70 to 90 degrees is over 6 times that of iron, about 5 times that of brass, and 4 times that of zinc which is the most susceptible of all metals to expansion by heat. It expands quite regularly up to 200 degrees, from this to the melting point 250 degrees, it expands excessively.

In vulcanizing, soon after chemical action begins (248 degrees) expansion ceases and contraction commences, continuing to a greater degree than the expansion in heating up.

### HOW TO VULCANIZE RUBBER.

Dr. Wilson by a series of experiments, the results of which he had with him, showed how essential proper vulcanization is to the welfare of the denture. The conclusions arrived at by these experiments:

The flask should be well warmed, but not above 212 degrees. The mould evenly packed with red or black rubber or a combination of the two, but must in no place be thicker than 3 mm. or at the outside 4 mm.; all space beyond that must be filled with pink or white rubber. The flask must be cautiously nearly closed under pressure, but the final closure should be in the vulcanizer under pressure from a volute spring attached to a Donham clamp. With this method no gateways are needed, but if the flask is bolted gateways opening into vent spaces are imperative.

Vulcanizing should be done in steam, and at high temperature and short time, that is, sufficient heat should be applied to reach the maximum temperature (320 degrees) in 25 minutes, not varying more than 5 minutes either way, then the high heat should be maintained for say 55 minutes.

Preparation of the case for flasking.—Strings are used for outlining the festoons and periphery of the gum. The object of the festooning string at the cervical portion of the teeth is to give the proper thickness to the margin of the gum. The string used for this purpose is waxed dental floss, twisted very hard, doubled and twisted again. Wax the string well with softened wax and apply it by grasping the left heel of the wax model between the fingers and thumb of the left hand, with the occlusal surface of the teeth upward; place one end of the string at the distal surface of the second molar, pressing it gently into the wax; outline the margin of the gum, using the wax spatula to carry the string well into the interproximal spaces.

The peripheral string should be well waxed, wrapping twine placed at the outer edge of the wax, and secured in place by melted wax made smooth with a hot spatula. The peripheral string should be applied at the line of separation of the flask and this must be, in cases of heavy restorations, at the widest portion of the wax model.

The next step is to cover the buccal and labial surfaces with a strip of number 60 tin foil. The number 3 instrument of the Evans set of carvers is especially adapted for adjusting the foil. The strip of tin foil, about a third of a sheet, is placed over the wax and teeth and pressed as closely as possible with the fingers. The surplus tin is trimmed to a little below the top of the teeth with a pair of scissors. The tin should be slit between each two teeth.

Hold the work in the left hand, seize the instrument by the hand grasp, rest the thumb upon the occlusal surface of the second molar, and burnish the tin closely to the tooth and against the festoon string.

Continue this operation with all the teeth. After adjusting the tin about the teeth the metal must be burnished over the string to give the desired thickness of the gum and the contour of the festoon. This is done by holding the plate and burnisher in the same manner as before.

The instrument must extend one-sixteenth of an inch beyond the string and at the same time must rest upon the body of the tooth while pressing the tin down over the festoon string. By this means a proper thickness and contour are given the margin of the gum without forming an unnatural beaded edge. After all the teeth have been thus treated, the position of the plate should be reversed in the left hand, so that the thumb of the right hand may rest upon the periphery of the base plate while burnishing the tin from the festoons toward the periphery, with a pair of sharp curved scissors trim the tin flush with the peripherical string, but do not permit it to overlap the vulcanite base plate.

The case is now ready for tinning the lingual surface, use number 60 foil, and if the vault is a high one slit the tin from the middle of one side to the center. Place the inner end of the slit over the middle of the vault, and one edge of the slit along the raphe to the palatal border; press the side of the foil against the wax and teeth; press the other half of the tin in the same manner, into position permitting the slit portion to overlap the first half. With sharp scissors trim the foil nearly down to the teeth. Remove the foil and place it upon a plaster or metal cast, having well defined rugae., and burnish the rugae into the foil. Remove the foil, turn it over, and fill the impression of the rugae with wax, also smear the remainder of this surface with a thin layer of wax; now replace the wax surface against the vault of the plate and nicely adjust with the fingers. The tin must be securely burnished against the teeth. The lingual contour of the teeth is produced by roughly carving away the excess wax, with the Evans wax carvers just before placing the tin. The tin is also slit between the teeth on the lingual aspect and nicely burnished to place.

Tin foil number 4 is used for covering the cast. A sheet is fitted over the cast with thumb and finger. It is then removed and excess tin cut away with sharp scissors, as indicated by the imprint of the edge of the cast upon the tin. The cast is now coated with sandrac varnish, and when it has dried to tackiness the tin is at once replaced and firmly pressed and gently rubbed with a wad of soft paper until there is perfect adaptation to the cast. The tin should be rubbed until it has a well burnished surface. It is important that the tinned cast be coated with a lather of soap. If the soap is not applied the tin and vulcanite will adhere very strongly, and can only be separated by dissolving the tin with mercury or hydrochloric acid.

### ESTHETICS.

Prosthetic dentistry, has two exceedingly important essential subdivisions; the mechanical and the esthetic. The mechanical refers to that part of the work performed in the laboratory, the esthetic to the harmonizing of our work or creation with its environment, or the art of concealing art. In order to practise prothesis it is necessary for the patient to be under the inspection and study of the prosthetist, so that he can restore the lost form and harmonize the associate parts. To be an esthetic prosthetist the face must be studied carefully and the teeth must be set and colored accordingly.

No profession has more use for the esthetic and beautiful than that of dentistry. Proper form and color are the two essentials in the selection of teeth. The knowledge of the former can be arrived at by studying the outline of the face, following Dr. Leon Williams' method, grinding and coloring those selected to match the conditions found. In reference to color, if the color can not be selected at the supply house the use of S. S. W.'s mineral stains will enable the prosthetist to color to suit himself.

We are told that as to contour there are three types of faces—straight, convex and concave.

Dr. Wilson, by means of lantern slides, showed pictures of many celebrated persons, pointing out how the different faces fell into line with the above classification. He also used lantern slides to show how beautifully the harmony of the features of the face had been restored to some of his patients by careful attention to esthetics.—*Oral Health.*

